# Antibacterial, Antifungal and Cytotoxic Isoquinoline Alkaloids from *Litsea cubeba*

**DOI:** 10.3390/molecules171112950

**Published:** 2012-11-01

**Authors:** Wei Zhang, Jin-Feng Hu, Wen-Wen Lv, Qing-Chun Zhao, Guo-Bing Shi

**Affiliations:** 1 Department of Pharmacy, General Hospital of Shenyang Military Area Command, Shenyang 110840, China; 2 Department of Pharmacology, Institute of Materia Medica, Chinese Academy of Medical Sciences and Peking Union Medical College, Beijing 100050, China

**Keywords:** *Litsea cubeba*, Lauraceae, isoquinoline alkaloids, antibacterial, antifungal, cytotoxicity

## Abstract

Five novel isoquinoline alkaloids (+)-*N-*(methoxylcarbonyl)-*N-*nordicentrin (**1**), (+)-*N-*(methoxylcarbonyl)-*N-*norpredicentrine (**2**), (+)-*N-*(methoxylcarbonyl)-*N-*norbulbodione (**3**), and (+)-*N-*(methoxylcarbonyl)-*N-*norisocorydione (**4**), and (+)-8-methoxyisolaurenine-*N-*oxide (**5**) were isolated, together with one known compound, (+)-*N-*(methoxylcarbonyl)-*N-*norglaucine (**6**), from a 70% EtOH extract of the barks of *Litsea cubeba.* Structural elucidation of all the compounds were performed by spectral methods such as 1D- and 2D-NMR, IR, UV, and HRESIMS. Alkaloids **1**, **2** and **6** showed antimicrobial activity against the bacterium *S. aureus* and two fungi (*A. alternata* and *C. nicotianae*). Compounds **3**,**4** exhibited significant cytotoxicity against all of six tested tumor cell lines.

## 1. Introduction

**Figure 1:**
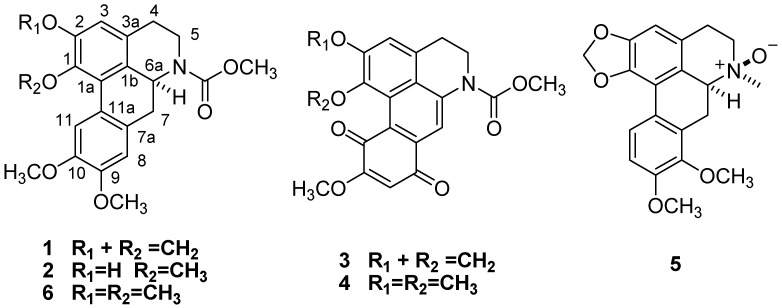
The structures of compounds **1**–**6**.

*Litsea cubeba* (Lauraceae ) is a 3- to 10 m evergreen tree or shrub widely distributed in Southeastern Asia, Southern China, Japan, and Taiwan [1,2]. *L. cubeba* can be used as a flavoring or herbal medicine. As a flavoring, it gives a unique flavor resembling that of a mixture of pepper, ginger, and citrus. It is used as a flavor enhancer in foods, cosmetics, and cigarettes [3,4]. The bark of *L. cubeba* has been used in oriental traditional medicine for the treatment of atopic eczema and coronary heart disease, and its antioxidant activities and antimicrobial activities against *Staphylococcus aureus*, *Salmonella typhi*, and *Pseudomonas aeruginosa* have been reported [5,6,7]. The essential oil from *L. cubeba* has good fungicidal activities against *Sclerotinia sclerotiorum*, *Thanatephorus cucumeris*, *Pseudocercospora musae* and *Colletotrichum gloeosporioides* [8,9].Various types of alkaloids have been isolated from this plant [10,11,12]. Litebamine and its *N-*homologues possess acetylcholinesterase activity [13]. Moreover, litebamine can inhibit platelet aggregation, adenosine 5'-triphosphate (ATP) release and thromboxane B2 formation induced by arachidonic acid and collagen in rabbit platelets [14]. In the present paper, chromatographic separation of an aqueous EtOH extract of the barks of *L. cubeba* has yielded five novel isoquinoline alkaloids, namely (+)-*N-*(methoxylcarbonyl)-*N-*nordicentrin (**1**), (+)-*N-*(methoxycarbonyl)-*N-*nor-predicentrine (**2**), (+)-*N-*(methoxyl-carbonyl)-*N-*norbulbodione (**3**), and (+)-*N-*(methoxycarbonyl)-*N-*nor-isocorydione (**4**), and (+)-8-methoxyl-isolaurenine *N-*oxide (**5**), and one known compound, (+)-*N-*(methoxycarbonyl)-*N-*norglaucine (**6**) (Figure 1). Their structures were established on the basis of their chromatographic properties, chemical and physicochemical methods. Furthermore, all the triterpenoids were evaluated for their *in vitro* antibacterial, antifungal and cytotoxic properties.

## 2. Results and Discussion

**Figure 2:**
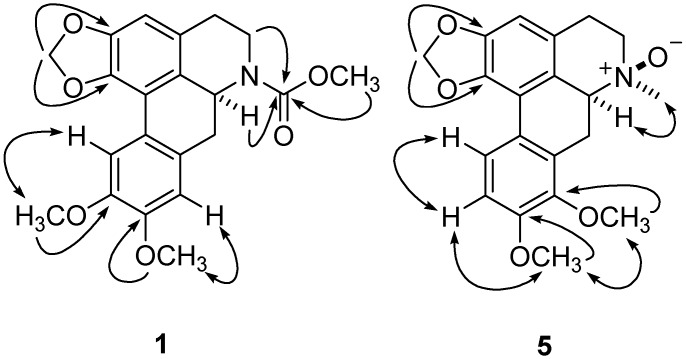
Key HMBC (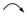) and ^1^H-^1^H COSY (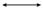) correlations of compounds **1** and **5**.

Compound **1** was obtained as a brown amorphous powder. The HRESIMS displayed a pseudomolecular ion at *m/z* 406.1263 [M+Na]^+^ (calcd for C_21_H_21_NO_6_Na, 406.1267) consistent with a molecular formula of C_21_H_21_NO_6_, corresponding to 12 degrees of unsaturation. Its UV absorption at λ_max_ 303, 284, and 216 nm suggested an aporphine alkaloid skeleton with substituents at C-1, C-2, C-9, and C-10 [15]. Its ^13^C-NMR spectrum showed 21 carbon signals [OCH_3_ × 3, OCH_2_O × 1, CH_2_ (sp^3^) × 3, CH (sp^3^) × 1, CH (sp^2^) × 3 and C (sp^2^) × 10, Table 1]. The ^1^H-NMR spectrum of **1** exhibited three aromatic singlets at δ_H_ 6.86 (H-3), 6.78 (H-8), and 8.16 (H-11), a methylenedioxy signal at δ_H_ 6.18, and three methoxys (δ_H_ 3.69, 3.90, and 3.93). The IR absorption at 1665 cm^–^^1^ and a signal at δ_C_ 157.2 in the ^13^C-NMR spectrum evidenced the presence of a carbamate moiety [16]. The position of the carbamate group at *N-*6 was supported by the HMBC correlations (Figure 2) of δ_C_ 157.2 with δ_H_ 3.69 (3H, s, OMe), 4.70 (1H, *dd*, *J* = 13.8, 3.8 Hz, H-6a), 4.42 (1H, *m*, H-5a), and 2.98 (1H, *m*, H-5b). Meanwhile, in the ^13^C-NMR spectra of **1**, signals of C-7 and -CO appeared as broad peaks due to the stereo-hindrance effect of methyl ester with C-7 on the NMR time scale at room temperature, and signals sharpened when measured under heating conditions (50 °C). The positions of two other methoxys were assigned based on the NOESY spectrum (Figure 2). The NOE correlations of H-8 and H-11 with the signals of two OCH_3_ (δ_H_ 3.90 and 3.93, respectively) positioned two methoxys at C-9 and C-10, which was further supported by the HMBC of C-9 (δ_C_ 147.2) with OCH_3_ (δ_H_ 3.90) and C-10 (δ_C_ 148.0) with OCH_3_ (δ_H_ 3.93) that the two OCH_3_ respectively (Figure 2). These data showed similarities to those of (+)-*N-*(methoxycarbonyl)-*N-*norglaucine (**6**). The stereochemistry of C-6a was determined to be *S* by its positive specific rotation (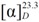 = +96.13) [17]. Therefore, compound **1** was identified as (+)-*N-*(methoxycarbonyl)-*N-*nordicentrin.

Compound **2** was obtained as a brown amorphous powder. The EIMS afforded a molecular weight of *m/z* = 385, and its HR-ESI-MS revealed the [M+Na]^+^ peak at *m/z* = 408.1422 (calcd. for C_21_H_23_NO_6_Na. 408.1423), corresponding to the molecular formula C_21_H_23_NO_6_. Comparing the ^1^H- and ^13^C-NMR data of **2** with those of compound **1**, the data were almost identical. The only significant difference was that the signals of the methylenedioxy moiety at C-1 and C-2 was replaced by a OH and OMe groups in compound **2**. The HMBC correlations of C-1 (δ_C_ 144.8) with OCH_3_ (δ_H_ 3.56) indicated the location of MeO and OH at C-1 and C-2 respectively, which was confirmed by the downfield shift of C-2 from δ_C_ 146.8 to 150.5. On the basis of the observation of NOESY data similar to those of **1** and the positive specific rotation (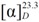 = +258.7 [17], the stereochemistry of **2** was expected to be the same. Accordingly, the structure of **2** was established as (+)-*N*-(methoxycarbonyl)-*N*-norpredicentrine.

Compound **3**, a violet amorphous powder, exhibited a molecular formula of C_20_H_15_NO_7_, based on the HRESIMS spectrum which showed a pseudomolecular ion at *m/z* 404.0743 [M+Na]^+^ (calcd. 404.0746). The ^l^H-NMR spectrum showed singlet signals for three aryl protons (δ_H_ 6.91, 6.88, and 5.92), two OMe (δ_H_ 3.71 and 3.85), one methylenedioxy (δ_H_ 6.20), and two triplets for two vicinal coupled methylenes [δ_H_ 2.63, 2.88 (H-4) and 2.99, 4.43 (H-5)]. The IR spectrum exhibited a conjugated carbonyl absorption (1654 cm^−1^), and the ^13^C-NMR spectrum displayed two carbonyl signals at δ_C_ 178.3 and 186.4, similar to the 1,4-dicarbonyl signals in *N-*norbulbodione [18]. The HMBC correlations of δ_C_ = 156.2 with δ_H_ 3.71 (3H, *s*, OMe), 2.99 (1H, *m*, H-5a), and 4.33 (1H, *m*, H-5b) indicated the presence of a *N-*carbamate group. The methylenedioxyl unit was positioned between C-1 and C-2, and the methoxyl at C-10, respectively, based on the HMBC correlations of the proton signal of methylenedioxy with C-1 and C-2, and of the methoxyl signal (δ_H_ 3.85) with C-10, respectively. The positive specific rotation (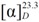 = +77.8 indicated stereochemistry of C-6a was determined to be *S* [19] Thus, Compound **3** was elucidated as (+)-*N-*(methoxycarbonyl)-*N-*norbulbodione.

**Table 1:** ^1^H-NMR data of compounds **1–5** in CDCl_3_ (δ in ppm and *J* in Hz).

No.	δ ^1^H (Hz)						δ ^13^C				
	1	2	3	4	5		1	2	3	4	5
1	-	-	-	-	-		142.8	144.8	141.7	143.8	142.7
1a	-	-	-	-	-		127.8	128.5	127.0	127.1	121.4
1b	-	-	-	-	-		129.9	131.6	116.0	116.1	122.1
2	-	-	-	-	-		146.8	150.5	145.5	151.9	146.6
3	6.86 (*s*)	6.56 (*s*)	6.88 (*s*)	6.63 (*s*)	6.69 (*s*)		107.5	115.3	107.3	111.0	109.6
3a	-	-	-	-	-		125.2	125.2	127.1	127.2	121.9
4	2.59, 2.84 (*m*)	2.52, 2.72 (*m*)	2.63, 2.88 (*m*)	2.62, 2.86 (*m*)	2.73, 3.53 (*m*)		30.1	30.8	30.2	30.4	25.4
5	2.98, 4.42 (*m*)	2.87, 4.25 (*m*)	2.99, 4.43 (*m*)	3.00, 4.44 (*m*)	3.57, 3.71 (*m*)		38.9	40.3	35.6	35.6	65.6
6a	4.70 (*dd*, 13.8, 3.8)	4.55 (*dd*, 13.8, 4.0)	-	-	4.40 (*dd*, 13.8, 3.8)		51.6	53.2	139.9	140.0	72.5
7	2.74, 2.84 (*m*)	2.62, 2.75 (*m*)	6.91 (*s*)	6.96 (*s*)	3.06, 3.18 (*m*)		34.9	35.4	98.2	98.3	30.2
7a	-	-	-	-	-		124.0	124.1	136.5	136.5	124.8
8	6.78 (*s*)	6.80 (*s*)	-	-	-		109.9	111.2	186.4	186.6	142.1
9	-	-	5.92 (*s*)	5.94 (*s*)	-		147.2	147.3	105.0	105.2	146.2
10	-	-	-	-	7.02 (*d*, 9.2)		148.0	148.2	163.8	163.9	109.6
11	8.16 (*s*)	8.16 (*s*)	-	-	8.66 (*d*, 9.2)		111.5	111.5	178.3	178.5	117.4
11a	-	-	-	-	-		129.6	129.9	117.9	118.1	128.5
1-OCH_3_	-	3.56 (*s*)	-	3.65 (*s*)	-		-	60.1	-	59.8	-
2-OCH_3_	-	-	-	3.90 (*s*)	-		-	-	-	55.7	-
8-OCH_3_	-	-	-	-	4.00 (*s*)		-	-	-	-	60.7
9-OCH_3_	3.90 (*s*)	3.91 (*s*)	-	-	3.98 (*s*)		55.8	55.8	-	-	56.2
10-OCH_3_	3.93 (*s*)	3.93 (*s*)	3.85 (*s*)	3.88 (*s*)	-		55.9	56.0	56.4	56.6	-
OCH_2_O	6.18 (*s*)	-	6.20 (*s*)	-	6.20 (*s*)		101.1	-	101.3		101.4
*C*O_2_CH_3_	-	-	-	-	-		157.2	157.6	156.2	156.0	-
CO_2_*CH_3_*	3.69 (*s*)	3.72 (*s*)	3.71 (*s*)	3.70 (*s*)	-		52.8	53.2	52.9	52.6	-
*N-CH_3_*	-	-	-	-	3.35 (*s*)		-	-	-	-	58.0

Compound **4** was obtained as a violet amorphous powder. Its positive HRESIMS spectrum showed a quasimolecular ion peak at *m/z* = 420.1055 [M+Na]^+^, consistent with the molecular formula C_21_H_19_NO_7_, accounting for 13 degrees of unsaturation. The general features of its IR and NMR spectra closely resembled those of **3**, except for the chemical shifts of two methoxyls in **4** taking the place of the methylenedioxyl between C-1 and C-2 in **3**, which was confirmed by HMBC correlations from the two methoxyl signal (δ_H_ 3.65 and 3.90) with C-1 (δ_C_ 143.8) and C-2 (δ_C_ 151.9), respectively. The stereochemistry of C-6a was established as *S* as inferred from its positive specific rotation (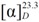 = +87.3) [19]. Thus, the structure of **4** was assigned the name (+)-*N-*(methoxycarbonyl)-*N-*norisocorydione.

Compound **5** exhibited a quasimolecular ion peak at *m/z* 378.1315 ([M+Na]*^+^*) in the high-resolution mass spectrometry, which corresponded to the molecular formula C_20_H_21_NO_5_. The UV spectrum showed absorptions at λ_max_ = 304, 284, and 217 nm, characteristic of a 1, 2, 8, 9-substituted aporphine. Its ^1^H-NMR spectrum showed a very similar pattern to those of (+)-isolaurenine *N-*oxide [16], including a methylenedioxyl (δ_H_ 6.20), two coupled aromatic doublets at δ_H_ 7.02 (1H, *d*, *J* = 9.2, H-10) and 8.66 (1H, *d*, *J* = 9.2, H-11), an aromatic singlets at δ_H_ 6.69 (*s*), two OCH_3_ singlets at δ_H_ 3.98 and 4.00, and three highly downfield shifts of *N-*CH_3_ at δ_H_ = 3.35 (3H, *s*), H-6a at δ_H_ 4.40(1H, *dd*, *J* = 13.8, 3.8 Hz), and H-5 at δ_H_ 3.57 and 3.71 (each 1H, *m*) due to the *N-*oxide. The HMBC correlations of the methylenedioxyl proton signal with C-1 and C-2, and of the two methoxyl signals (δ_H_ 3.98 and 4.00) with C-8 and C-9, respectively, indicated that methylenedioxyl were located between C-1 and C-2, and the methoxyls at C-8 and C-9 respectively. The NOE correlations of H-10 with H-11, and H-11 with OCH_3_ (δ_H_ 3.98, *s*) further supported the position of the methoxyl groups at C-8 and C-9. The positive specific rotation (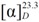 = +88.2) of **5** indicated an *S* configuration of C-6a [17], compared with the *R* configuration of that in (−)-isoboldine *β*-*N-*oxide (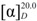 = −90.3) [20]. Furthermore, the key NOE correlations of *N-*CH3 with H-6 indicated the β-*N-*oxide in **5** (Figure 2). Thus, compound **5** was determined to be (+)-8-methoxyisolaurenine *N-*oxide.

All compounds were tested for their antimicrobial activities the by disc diffusion method by measuring the inhibition zones and for the most active compounds, minimum inhibitory concentration (MIC) values were also determined. Interesting antimicrobial properties were observed (Table 2), showing that alkaloids **1**, **2** and **6** exhibited antimicrobial activity against the bacterium *S. aureus* and two fungi (*A. alternata* and *C. nicotianae*), with MIC values of 0.60–0.80 mM, 0.74–1.04 mM and 1.41–2.14 mM, respectively. Compounds **3**,**4** have antibacterial activities against *S. aureus*, while compound 5 showed weak activity against the fungus *A. alternata*. Moreover, alkaloids **1**–**4** and **6** possessed higher antibacterial, antifungal activities with lower MIC value than **5**. These result suggested that the *N-*carbamate group may strengthen the antibacterial and antifungal activities of this type of alkaloids.

The *in vitro* cytotoxic activities of the isolated alkaloids were determined against BGC-823 cells (human gastric carcinoma), HepG2 cells (Human hepatocellular carcinoma), MCF-7 cells (human breast cancer), SGC-7901 cells (human gastric adenocarcinoma), SK-MEL-2 (human skin cancer), and with SK-OV-3 (ovarian) using the revised MTT method. The results are summarized in Table 3. Among the tested compounds, alkaloids **3**,**4** with two carbonyl groups at C-8 and C-11 exhibited the most potent cytotoxicity against all tested tumor cell lines, with IC_50_ values of 9.54–12.22 μM and 9.83–11.96 μM, respectively. Compounds **1**, **2** and **6** showed moderate cytotoxicity against the six tumor cell lines, while **5** had the weakest activities, with an IC_50_ value above 70 μM.

**Table 2:** Antimicrobial and antifungal activities (zones of inhibition/and MIC mM, n = 3) of compounds **1**–**6**.

Compound	*S.aureus*	*M. tuberculosis*	*G. pulicaris*	*A. alternata*	*C. nicotianae*	*P. capsici.*	*G. amomi*
**1**	20/0.68	-	-	19/0.64	18/0.80	-	-
**2**	22/0.79	-	-	20/0.74	17/1.04	-	-
**3**	16.53	-	-	-	-	-	-
**4**	17.62	-	-	-	-	-	-
**5**	-	-	-	15.35	-	-	-
**6**	17/2.14	-	-	19/1.41	16/1.70	-	-
Rifampicin	25/0.003	22/0.003	-	-	-	-	-
Nystatin	-	-	20/0.008	17/0.007	21/0.006	18/0.061	19/0.010

-: No activity.

**Table 3:** Cytotoxicity of compounds **1**–**6** against six human tumor cell lines (IC_50_, μM).

Compound	Cell lines					
	BGC-823	HepG2	MCF-7	SGC-7901	SK-MEL-2	SK-OV-3
**1**	31.87	28.09	30.13	29.49	29.70	29.45
**2**	30.08	30.48	29.68	30.88	27.09	30.10
**3**	10.38	9.54	11.65	10.34	11.44	12.22
**4**	9.83	10.38	10.81	11.86	10.59	11.96
**5**	83.22	86.62	78.23	76.87	85.03	92.97
**6**	31.51	33.78	30.72	28.94	33.12	31.84
Doxorubicin	0.02	0.01	0.06	0.05	0.03	0.01

## 3. Experimental

### 3.1. General

Melting points were determined using a Fisher-Johns melting point apparatus (Vernon Hills, Lake, IL, USA). Optical rotations were determined with a JASCO P2000 digital polarimeter (Tokyo, Japan). Ultraviolet (UV) and infrared (IR) spectra were obtained on JASCO V-650 and JASCO FT/IR-4100 spectrophotometers, respectively. The NMR spectra were recorded on a Varian Unity INOVA 600 FT-NMR spectrometer ((Salt Lake City, UT, USA; 600 MHz for ^1^H; 125 MHz for ^13^C, respectively). Chemical shifts were reported using residual CDCl_3_ (δ_H_ 7.26 and δ_C_ 77.0 ppm) and CD_3_OD (δ_H_ 3.30 and δ_C_ 49.0 ppm) as internal standard. High resolution ESIMS spectra were obtained on a LTQ Orbitrap XL (Thermo Fisher Scientific, Waltham, MA, USA) spectrometer. Silica gel 60 (Merck, Darmstadt, Germany, 230–400 mesh), LiChroprep RP-18 (Merck, 40–63 μm), and Sephadex LH-20 (Amersham Pharmacia Biotech., Roosendaal, The Netherlands) were used for column chromatography (CC). Precoated silica gel plates (Merck, Kieselgel 60 F254, 0.25 mm) and precoated RP-18 F_254s_ plates (Merck) were used for analytical thin*-*layer chromatography analyses.

### 3.2. Plant Material

The barks of *L. cubeba* was collected in the Tongren, Guizhou Province, China, in July 2011. A specimen (201107001A) was identified by one of the authors (Q.C. Zhao) and deposited in the Herbarium of Shengyang Medicine College, Shengyang, China.

### 3.3. Extraction and Isolation

The barks of *L. cubeba* (9.6 kg) were cut into small pieces and were extracted with 70% EtOH (20 L × 3) at room temperature for 24 h each time. After removal of EtOH under reduced pressure at 55 °C, the aqueous brownish syrup (1 L) was suspended in H_2_O (l L) and then succesively partitioned with petroleum ether (1 L × 3), chloroform (1 L × 3), and *n-*butanol (1 L × 3) to afford fractions of 43.2 g, 57.7 g, and 73.2 g, respectively. The chloroform fraction was further fractionated through a silica gel column (200–300 mesh, 10 × 80 cm, 500 g) using increasing volumes of acetone in petroleum ether (b.p. 60–90 °C) (100:1, 50:1, 30:1, 15:1, 10:1, 7:1, 5:1, 3:1, 1:1, v/v) as the eluent to give 10 fractions according to TLC analysis. Fraction 4 (petroleum ether-acetone 15:1, 3.6 g) was applied to an ODS MPLC column (100 g) and eluted with MeOH-H_2_O (20:80, 30:70, 40:60, each 500 mL) to yield four subfractions (Fr. 4-1 and Fr. 4-4). Subfraction 4-2 (MeOH-H_2_O, 350 mg) was purified by preparative RP-HPLC (ODS column, 250 × 20 mm) using MeOH-H_2_O (25:75) as mobile phase to obtain **1** (71 mg). Subfraction 4-2 (MeOH-H_2_O, 350 mg) was chromatographed by a Sephadex LH-20 column eluted with MeOH-H_2_O (50:50), and purifed by preparative RP-HPLC (ODS column, 250 × 20 mm) using MeOH-H_2_O (30:70) as mobile phase to yield **3** (88 mg) and **6** (75 mg). Subfraction 4-4 (MeOH-H_2_O 40:60, 99 mg) was purified by preparative RP-HPLC (ODS column, 250 × 20 mm) eluting with MeOH/H_2_O (22:78) to get **4** (57 mg). Fraction 5 (petroleum ether-acetone 30:1, 1.3 g) was applied to an ODS column eluted with MeOH-H_2_O (30:70, 40:60, 50:50) to provide three subfractions (Fr. 5-1 and Fr. 5-3), Subfraction 5-2 (MeOH-H_2_O 20:80, 226 mg) was was repeatedly chromatographed on silica gel (150 g, 60 × 2.8 cm, chloroform-methanol, 20:1 → 10:1) and then purifed on a Sephadex LH-20 column eluted with MeOH-H_2_O (50:50) to afford **2** (78 mg). Subfraction 5-3 was purified by preparative RP-HPLC (*ODS* column, 250 × 20 mm) eluting with MeOH/H_2_O (20:80) to get **5** (77 mg).

*(+)-N-(Methoxycarbonyl)-N-nordicentrine* (**1**): brown amorphous powder. 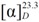 = +96.13 (*c* = 0.19, MeOH). UV (CDCl_3_) λ_max_(log*ε*) 303 (4.12), 284 (3.90), 216 (3.89) nm. IR (KBr)*ν*_max_ 3030, 1705, 1665, 1452, 1255 cm^−1^. ^1^H-NMR (CDCl_3_, 600 MHz) and ^13^C-NMR (CDCl_3_, 125 MHz) data see Table 1. EI-MS *m/z*: 383 ([M]^+^). HR-ESI-MS (pos.) *m/z*: 406.1263 ([M+Na]^+^, C_21_H_21_NO_6_Na. calc. 406.1267).

*(+)-N-(Methoxycarbonyl)-N-norpredicentrine* (**2**): brown amorphous powder. 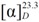 = +258.7 (*c* = 0.16, MeOH). UV (CDCl_3_) λ_max_(log*ε*) 304 (3.73), 283 (4.20), 216 (3.99) nm. IR (KBr)*ν*_max_ 3430, 1670, 1600, 1525, 1208 cm^−1^. ^1^H-NMR (CDCl_3_, 600 MHz) and ^13^C-NMR (CDCl_3_, 125 MHz) data see Table 1. EI-MS *m/z*: 385 ([M]^+^). HR-ESI-MS (pos.) *m/z*: calc. 408.1422 ([M+Na]^+^, C_21_H_23_NO_6_Na. calc. 408.1423).

*(+)-N-(Methoxycarbonyl)-N-norbulbodione* (**3**): violet amorphous powder. 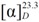 = +77.8 (*c* = 0.21, MeOH). UV (CDCl_3_) λ_max_(log *ε*) 304 (4.11), 284 (3.81), 217 (4.11) nm. IR (KBr)*ν*_max_ 3028, 1707, 1654, 1455, 1251 cm^−1^. ^1^H-NMR (CDCl_3_, 600 MHz) and ^13^C-NMR (CDCl_3_, 125 MHz) data see Table 1. EI-MS *m/z*: 381 ([M]^+^). HR-ESI-MS (pos.) *m/z*: 404.0743 ([M+Na]^+^, C_20_H_15_NO_7_Na. calc. 404.0746).

*(+)-N-(Methoxycarbonyl)-N-norisocorydione* (**4**): violet amorphous powder. 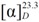 = +87.3 (*c* = 0.16, MeOH). UV (CDCl_3_) λ_max_(log*ε*) 305 (4.25), 283 (3.86), 215 (3.98) nm. IR (KBr)*ν*_max_ 3025, 1710, 1510, 1454, 1245 cm^−1^. ^1^H-NMR (CDCl_3_, 600 MHz) and ^13^C-NMR (CDCl_3_, 125 MHz) data see Table 1. EI-MS *m/z*: 397 ([M]^+^). HR-ESI-MS (pos.) *m/z*: 420.1055 ([M+Na]^+^, C_21_H_19_NO_7_Na. calc. 420.1059).

*(+)-8-Methoxisolaurenine N-oxide* (**5**): brown amorphous powder. 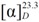 = +88.2 (*c* = 0.20, MeOH). UV (CDCl_3_) λ_max_(log *ε*) 304 (3.69), 284 (3.77), 217 (4.07) nm. IR (KBr)*ν*_max_ 3030, 1568, 1213, 1075, 1025 cm^−1^. ^1^H-NMR (CDCl_3_, 600 MHz) and ^13^C-NMR (CDCl_3_, 125 MHz) data see Table 1. EI-MS: 355 ([M]^+^). HR-ESI-MS (pos.) *m/z*: 378.1315 ([M+Na]^+^, C_20_H_21_NO_5_Na. calc. 378.1317).

### 3.4. Antimicrobial Activity Bioassay

All compounds (purity > 90%) were screened for their antimicrobial activity *in vitro* using the disk-diffusion method as described in the literature with minor modifications [19]. Strains including two species of bacteria [*Staphylococcus aureus* (ATCC-25923), *Mycobacterium tuberculosis* (ATCC-25177/H37Ra)] and five species of fungi [*Gibberella pulicaris* (KZN 4207), *Alternaria alternata* (TX-8025), *Colletotrichum nicotianae* (SACC-1922), *Phytophthora capsici* (KACC-40157), *Gonatopyricularia amomi* (MB-9671)] were used. Rifampicin and nystatin were used as positive controls for antibacterial and antifungal activities, respectively. A disk containing only DMSO was used as the negative control. Medium used in the antimicrobial activity included nutrient agar medium (*S. aureus*), Dorset egg medium (*M. tuberculosis*) and Sabouraud dextrose broth (SDB) agar medium (five species of fungi). To each agar plate, an inoculum containing 10^7^ bacteria/mL or a 0.5 optical density of the McFarland Scale was incorporated. The plates were solidified and sterile filter paper disks (6-mm diameter) were done on each one. Solution of each compound (5 mM) in DMSO, antibacterial agents (rifampicin 5 μM/mL), antifungal agents (nystatin 10 μM/mL), control vehicles (DMSO) were added into too. The plates were aerobically incubated at 37 °C for *S. aureus* during 18 h, for the five species of fungi during 24 h and for *M. tuberculosis* during 15–45 days, and four assays under identical conditions were carried out for each one. The diameter of the inhibition zone was measured for testing of antibacterial and antifungal activities. Experiments were performed in triplicate, and the results are presented as the mean values of the diameters of the inhibitory zones from three runs. The MIC values of the most active compounds, in the previous experiment, were determined using the dilution method in 96-hole plates. The diameters of the inhibitory zones and the MIC value were used as criteria to judge the antimicrobial activity (active: the diameters of the inhibitory zones ≥16 mm, MIC ≤ 5 mM; moderately active: the diameters of the inhibitory zones are visible, MIC > 5 mM; not active: the diameters of the inhibitory zones are invisible). All strains of bacteria and fungi were purchased from Shanghai Institute of Biochemistry & Cell Biology, Chinese Academy of Sciences (Shanghai, China).

### 3.5. Cytotoxicity Assay *in Vitro*

The cytotoxic activities of the isolated compounds were determined using the revised MTT method [21,22] against BGC-823 cells (human gastric carcinoma), HepG2 cells (Human hepatocellular carcinoma), MCF-7 cells (human breast cancer), SGC-7901 cells (human gastric adenocarcinoma), SK-MEL-2 (human skin cancer), and with SK-OV-3 (ovarian), with doxorubicin (DOX, adriamycin, Sigma Chemical Co., St. Louis, MO, USA) as positive control. Cancer cells (4 × 10^3^ cells) suspended in 100 μL/well of DMEM medium containing 10% fetal calf serum were seeded onto a 96-well culture plate. After 24 h pre-incubation at 37 °C in a humidified atmosphere of 5% CO_2_/95% air to allow cellular attachment, various concentrations of test solution were added and cells were incubated for 48 h under the above conditions. At the end of the incubation, 10 μL of tetrazolium reagent was added into each well followed by further incubation at 37 °C for 4 h. The supernatant was decanted, and DMSO (100 μL/well) was added to allow formosan solubilization. The optical density (OD) of each well was detected using a microplate reader at 550 nm and for correction at 595 nm. Each determination represented the average mean of six replicates. The 50% inhibition concentration (IC_50_ value) was determined by curve fitting and was used as criteria to judge the cytotoxicity (active: IC_50_ ≤ 20 μM; moderately active: 20 μM < IC_50_ ≤ 70 μM; not active: IC_50_ > 70 μM). All cell lines were purchased from Cell Bank of Shanghai Institute of Biochemistry & Cell Biology, Chinese Academy of Sciences. Other reagents were purchased from Shanghai Sangon Biological Engineering Technology & Services Co., Ltd. (Shanghai, China)

## 4. Conclusions

Phytochemical investigation of the 70% EtOH extract of *L. cubeba* led to the isolation of five novel isoquinoline alkaloids: (+)-*N-*(methoxycarbonyl)-*N-*nordicentrin (**1**),(+)-*N-*(methoxycarbonyl)-*N-*nor-predicentrine (**2**), (+)-*N-*(methoxycarbonyl)-*N-*norbulbodione (**3**), (+)-*N-*(methoxycarbonyl)-*N-*nor-isocorydione (**4**) and (+)-8-methoxyisolaurenine *N-*oxide (**5**) and one known compound, (+)-*N-*(methoxycarbonyl)-*N-*norglaucine (**6**). All the alkaloids were evaluated for their *in vitro* antimicrobial activities against two species of bacteria and five species of fungi and cytotoxic properties against BGC-823 cells (human gastric carcinoma), HepG2 cells (Human hepatocellular carcinoma), MCF-7 cells (human breast cancer), SGC-7901 cells (human gastric adenocarcinoma), SK-MEL-2 (human skin cancer), and with SK-OV-3 (ovarian). In the antimicrobial activity screening alkaloids **1**, **2** and **6** exhibited activity against the bacterium *S. aureus* and two fungi (*A. alternata* and *C. nicotianae*). Compounds **3**, **4** have antibacterial activities against *S. aureus*, while compound **5** showed weak activity against the fungus *A. alternata*. In the cytotoxicity bioassays, alkaloids **3**,**4** withcarbonyl groups at C-8 and C-11 exhibited the most potent cytotoxicity against all tumor cell lines, and compounds **1**,**2** and **5**,**6** showed rather moderate cytotoxicity against the six tumor cell lines.
